# Functional RNA Elements in the Dengue Virus Genome

**DOI:** 10.3390/v3091739

**Published:** 2011-09-15

**Authors:** Leopoldo G. Gebhard, Claudia V. Filomatori, Andrea V. Gamarnik

**Affiliations:** Fundación Instituto Leloir-CONICET, Avenida Patricias Argentinas 435, C1405BWE, Buenos Aires, Argentina; E-Mails: lgebhard@leloir.org.ar (L.G.G); cfilomatori@leloir.org.ar (C.V.F.)

**Keywords:** flavivirus RNA, dengue virus, viral RdRp, genome cyclization, RNA structures, viral RNA replication, NS5 protein

## Abstract

Dengue virus (DENV) genome amplification is a process that involves the viral RNA, cellular and viral proteins, and a complex architecture of cellular membranes. The viral RNA is not a passive template during this process; it plays an active role providing RNA signals that act as promoters, enhancers and/or silencers of the replication process. RNA elements that modulate RNA replication were found at the 5′ and 3′ UTRs and within the viral coding sequence. The promoter for DENV RNA synthesis is a large stem loop structure located at the 5′ end of the genome. This structure specifically interacts with the viral polymerase NS5 and promotes RNA synthesis at the 3′ end of a circularized genome. The circular conformation of the viral genome is mediated by long range RNA-RNA interactions that span thousands of nucleotides. Recent studies have provided new information about the requirement of alternative, mutually exclusive, structures in the viral RNA, highlighting the idea that the viral genome is flexible and exists in different conformations. In this article, we describe elements in the promoter SLA and other RNA signals involved in NS5 polymerase binding and activity, and provide new ideas of how dynamic secondary and tertiary structures of the viral RNA participate in the viral life cycle.

## 1. Introduction: DENV Life Cycle

Dengue virus (DENV) is a member of the *Flavivirus* genus of the *Flaviviridae* family [1]. The *Flavivirus* genus includes other important human pathogens such as yellow fever (YFV), West Nile (WNV), Japanese encephalitis (JEV), and tick borne encephalitis (TBEV) [1]. Flaviviruses are enveloped viruses with a single stranded, ∼11 kb, positive-sense RNA genome. The genome encodes a single long open reading frame (ORF), flanked by highly structured 5′ and 3′ untranslated regions (UTRs).

**Figure 1.**
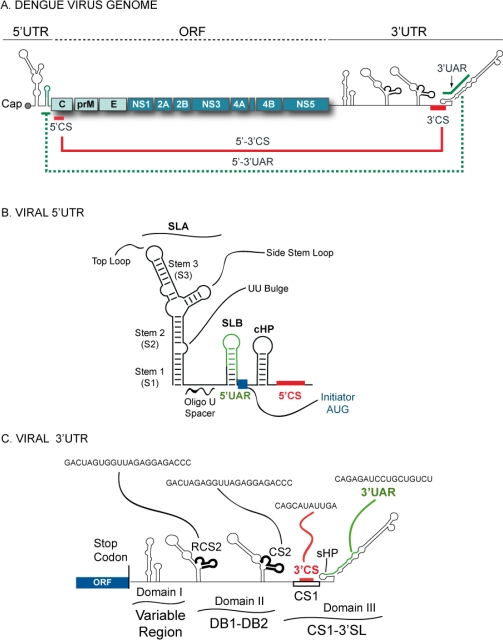
Schematic representation of the Dengue virus (DENV) genome. (**a**) The viral 5′ and 3′ untranslated regions (UTRs) and the open reading frame indicating structural proteins (C-prM-E) and non-structural proteins (NS1-NS2AB-NS3-NS4AB-NS5). The location of the complementary sequences 5′-3′CS and 5′-3′UAR are also indicated by solid and dashed lines, respectively. (**b**) Predicted secondary structure of the 5′ terminal region of the DENV genome. Structural elements located at the 5′ end: stem loop A (SLA), stem loop B (SLB), oligo(U) track spacer, translation initiator AUG, capsid region hairpin (cHP), and the 5′CS element. (**c**) Schematic representation of predicted RNA elements at the 3′UTR of the DENV genome. The predicted secondary structures of the three defined domains are indicated: domain I (variable region, VR), domain II (dumbbell structures, DB1 and DB2), and domain III (conserved sequence CS1 and 3′SL). In addition, the location and sequence of each of the conserved elements corresponding to RCS2, CS2, 3′CS, and 3′UAR are shown.

The virus enters the host cell by receptor mediated endocytosis. Upon internalization and acidification of the endosome, fusion of viral and vesicular membranes allows release of the genomic RNA into the cytoplasm, which serves as mRNA. Translation of the single ORF at the rough ER produces a large polyprotein that is cleaved co- and posttranslationally into the mature proteins. The N-terminal of the polyprotein encodes the three structural proteins (C-prM-E), followed by at least seven non-structural (NS) proteins (NS1-NS2A-NS2B-NS3-NS4A-NS4B-NS5) (Figure 1A) [2].

After translation of the RNA, virus-induced hypertrophy of intracellular membranes occurs, originating structures known as convoluted membranes and vesicle packets [3–5]. Flavivirus RNA synthesis occurs in close association with cellular membranes inside the vesicle packets in so-called viral replication complexes. The process begins with the synthesis of a negative strand RNA, which serves as template for the amplification of additional positive strand genomic RNA. The enzymatic reaction is catalyzed by the RNA-dependent RNA polymerase (RdRp) activity of the viral NS5 protein, in association with the viral protease/helicase NS3, other viral NS proteins, and presumably host factors. The newly synthesized RNA associates to the capsid (C) protein by a mechanism still unknown. The RNA-C complex buds into the ER lumen acquiring the lipid bilayer, and the viral E and prM proteins. Furin-mediated proteolysis of prM in the trans-Golgi network [6] triggers rearrangement, homodimerization of E, and formation of new viral particles [7].

## 2. RNA Structures in the DENV Genome

The DENV genome is 11 kb long, has a type 1 cap (m7GpppAmp) structure at the 5′ end, and lacks a poly(A) tail at the 3′ end. Besides encoding viral proteins, the genome contains RNA structures that regulate different viral processes. Upon infection, the incoming genome serves as mRNA for translation, and subsequently, as template for RNA synthesis. The newly synthesized RNA can be used for new rounds of translation or as substrate for encapsidation. Efficient utilization of the genome during these processes must be temporally regulated to ensure viral spread. This regulation is mediated by RNA elements present in the coding and non-coding regions of the viral genome acting as promoters, enhancers, and repressors of the viral processes. In addition, during infection the viral RNA participates in triggering or avoiding the antiviral host response [8,9].

DENV 5′UTRs are between 95 to 101 nucleotides long. They contain two RNA domains with distinct functions during viral RNA synthesis. The first domain of ∼70 nucleotides is predicted to fold into a large stem-loop (SLA, Figure 1B). A similar structure is present at the 5′UTR of other members of the Flavivirus genus [10–14]. DENV SLA has been proposed to act as the promoter for the viral RdRp (NS5). Direct binding of NS5 to SLA was shown to be necessary for viral RNA synthesis [15,16]. The second domain of the DENV 5′UTR is predicted to form a short stem loop (SLB), which contains essential sequences for long range RNA-RNA interaction and genome replication [17]. The two domains are separated by an oligo(U) sequence, which functions as spacer for proper function of the two stem loops (Figure 1B) [14]. The SLA is predicted to have a Y shaped structure, which was recently confirmed by enzymatic and chemical probing [14–18]. These studies indicate the presence of three helical regions (S1, S2, and S3) interrupted by bulges and highly reactive single stranded regions, in agreement with the presence of a side stem loop and a top loop (Figure 1B) [14]. The conserved structural elements described within the SLA of DENV are also found at the 5′ end of other members of the flavivirus genus (reviewed in [19]). In an initial study by Brinton and Dispoto, the 5′UTR sequences of different mosquito-borne flaviviruses were compared [12]. This study indicated that conserved secondary structures were present at the 5′ end of West Nile virus (WNV), Saint Louis encephalitis virus (SLEV), DENV, yellow fever virus (YFV), and Murray Valley encephalitis virus (MVEV) [12]. More recently, the predicted structures at the 5′ end of the genomes of tick-borne flaviviruses and flaviviruses with no known vector were found to be similar to that observed in the mosquito-borne flavivirus [13,20,21]. Within the coding sequence, just downstream of the AUG translation initiation codon, a stable hairpin (cHP, Figure 1B) was found in the DENV genome to be required for viral RNA replication [22].

Specific structures at the 3′ end of the viral genome also play crucial roles in viral RNA synthesis. The approximately 450 nucleotide long DENV 3′UTR can be divided into three domains (Figure 1C). Domain I is located immediately after the stop codon [23], and is the most variable region within the viral 3′UTR (VR). It exhibits extensive size variation between serotypes; ranging from more than 120 nucleotides to less than 50 nucleotides [24–29]. Domain II includes a characteristic dumbbell (DB) structure, which is duplicated in tandem (Figure 1C) [24–26]. The DB elements contain conserved sequences named CS2 and RCS2 (repeated CS2) present in all mosquito-borne flaviviruses [30–33]. In addition, sequences within the DB elements were proposed to be involved in two pseudoknot structures [34]. A recent report indicated a functional role of the pseudoknot structure during viral translation and RNA synthesis [35]. Although RNA elements within domains I and II are considered to be dispensable for flavivirus replication, these structures serve as enhancers of viral processes [23,36–39]. Domain III is the most conserved region of the 3′UTR, bearing a CS1 element followed by a terminal stem-loop structure (3′SL). CS1 contains a sequence involved in long range RNA-RNA interaction between the ends of the viral genome [30]. The 3′ terminal structure contains a short stem loop of 14 nucleotides (sHP) followed by a large stem loop of 79 nucleotides. The two adjacent structures involve 93 nucleotides and are referred to as 3′SL (Figure 1C). The existence and the essential role of the 3′SL have been supported by secondary structure predictions, co-variation analysis, biochemical probing, and functional studies in DENV and other members of the Flaviviridae family [2,16,30,40–48].

**Figure 2.**
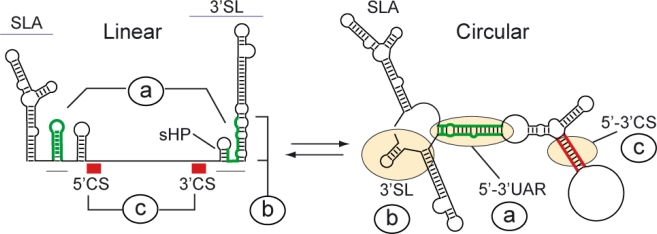
Schematic representation of predicted conformational changes in the transition between the linear and circular conformation of the DENV genome. Predicted changes of conserved RNA structures upon 5′-3′ end hybridization are indicated: a) the SLB and the sHP of the 3′SL observed in the linear conformation of the RNA open to form an extended duplex in the circular form, b) the large stem of the 3′SL observed in the linear form opens, releasing the last nucleotides of the genome in the circular form, and c) the complementary sequences 5′ and 3′ CS interact in the circular form to generate a double stranded region.

A conserved feature of DENV and other flavivirus genomes is the presence of inverted complementary sequences at the ends of the RNA that mediate long-range RNA-RNA interactions [10,11,17,30,49–51]. The significance of genome cyclization during viral replication is now beginning to be uncovered. At least two pairs of complementary regions and adjacent nucleotides are necessary for DENV genome cyclization (Figure 1A) (reviewed in [52]). These regions are known as 5′-3′CS and 5′-3′UAR, of 11 and 16 nucleotide long, respectively, and the adjacent DAR sequence [53]. The 5′CS is located inside the ORF, encoding amino acids 13 to 16 of the N-terminus of the capsid protein, and the 3′CS is located upstream of the highly conserved 3′SL (Figure 2). The 5′UAR is in the 5′UTR, just upstream of the translation initiator AUG, and the 3′UAR is located within the 3′SL, overlapping both the sHP and the bottom half of the large stem. The DAR region located between 5′UAR and 5′CS at the 5′, overlaps with the sHP within the 3′SL. Hybridization of the ends of the DENV genome results in conformational changes within conserved RNA structures: (a) the SLB and the sHP of the 3′SL open to form a duplex, (b) the large stem of the 3′SL opens, and (c) the sequences 5′ and 3′ CS form a double stranded region (Figure 2). Visualization of the DENV genome using atomic force microscopy (AFM) demonstrated cyclization of individual molecules by long range RNA-RNA interaction [17].

Studies from many different laboratories using infectious clones and replicon systems provided compelling evidence for the essential role of genome cyclization during flavivirus replication [17,39,49,54–57]. Mismatches within complementary regions did not alter translation of the viral RNA but greatly decreased RNA synthesis, leading in some cases to undetectable levels of viral replication. Compensatory mutations that restored 5′-3′ base pairing rescued RNA synthesis, indicating a role of RNA-RNA complementarity rather than the nucleotide sequence *per se* for viral replication [17,55,58].

More recently, it has been shown that the DENV genome is a dynamic molecule that acquires different conformations during viral replication [59]. In this regard, a balance between linear and circular forms of the genome (as described in detail below) has been found to be crucial for viral replication.

## 3. Functional Significance of DENV Genome Cyclization

An essential role of genome cyclization for replication of DENV and other flaviviruses has been demonstrated (for review see [52]). Molecular details that explain the requirement of genome cyclization were obtained by dissecting RNA elements involved in viral polymerase recognition. Studies performed by Padmanabhan and collaborators provided the first evidence that both ends of the DENV genome were necessary for RNA synthesis [52]. Using different regions of the DENV genome, the SLA structure present at the viral 5′UTR was found to be essential for specific NS5 polymerase binding and activity [15]. The location of the promoter element at the 5′ end of the genome was unexpected because the site for initiation of RNA synthesis is the 3′ end of the viral RNA. These findings indicated communication between the 5′ and 3′ ends of the genome during the RNA amplification process. The current model for DENV minus strand RNA synthesis includes NS5 binding at the 5′ end of the genome and relocation of the polymerase at the 3′ initiation site by genome cyclization mediated by long range RNA-RNA interactions [15].

Using *in vitro* polymerase assays with recombinant proteins, it has been demonstrated that an RNA molecule carrying the SLA was able to promote RNA synthesis by NS5 only if templates up to 500 nucleotides in length were used. It was proposed that the polymerase was unable to reach the 3′ end initiation site of longer RNA molecules. However, RNAs of about 2 kb carrying the 5′ and 3′ end viral sequences, which resemble the viral genome, were efficient templates for polymerase activity provided that the cyclization sequences were intact. This observation supported the notion that long range 5′-3′ RNA-RNA interactions in the template are necessary for the polymerase to reach the 3′ end of long RNA molecules. Mutagenesis analysis in the context of DENV infectious clones confirmed the requirement of both the SLA structure and the complementary sequences for viral RNA replication in cell culture. A correlation between the RNA structural elements required for polymerase activity *in vitro* and viral RNA replication in infected cells was observed, confirming the central role of the SLA promoter in viral RNA synthesis [14,15].

While it is not surprising to find a core promoter for RNA synthesis at the 3′ end of a viral genome, it is still intriguing why certain plus strand RNA viruses, such as DENV, would have promoters or enhancer elements for RNA replication at the 5′ end of the genome. Location of *cis*-acting RNA signals along the genome, and in some cases, thousands of nucleotides away from their site of action, indicates high flexibility of viral RNA molecules.

## 4. Elements of the SLA Promoter for NS5 Binding and Polymerase Activity

NS5 is the largest and the most conserved of the flavivirus proteins. It contains an N-terminal methyl transferase domain (MTase) and a C-terminal RdRp domain. The structure of the NS5 C-terminal domain of DENV revealed a classical polymerase fold, bearing palm, thumb, and finger motifs [60]. The presence of a priming loop found in this structure is consistent with *in vitro* data indicating a primer-independent (*de novo*) mechanism of initiation of flavivirus polymerases [61–63].

The viral protein NS5 has the ability to bind RNA with high affinity. Using mobility shift and filter binding assays, specific interaction of the full length NS5 and the RdRp domain of DENV with the 5′ terminal region of the viral genome was observed [15,64]. These proteins form specific ribonucleoprotein complexes with apparent dissociation constants (Kd) of 14 and 12 nM, for NS5 and the RdRp, respectively. Binding of the MTase domain to the viral RNA was also observed but with low affinity (Kd > 240 nM), indicating that DENV NS5 binds to the promoter SLA mainly through the RdRp domain [64,65].

Alignments of the SLA sequence from different flaviviruses together with probing analysis, indicated the presence of conserved structural RNA elements [14]. The SLA contains three helical regions (S1, S2, and S3), a top loop, and a side stem loop (Figure 1B). The S1 and S2 regions represent one of the most conserved elements within the flavivirus 5′UTRs [10]. In contrast, the sequence and structure of S3 and the side stem loop show the most variations. Recent reports have investigated the role of each of the conserved elements within the SLA promoter. Mutations within the SLA structure in the context of full length viral RNAs allowed the identification of sequences and structures necessary for viral replication [14,15].

Infectious DENV RNAs with mutations at each side of stems S1 and S2, disrupting stem formation, showed impaired replication. Revertant viruses carrying spontaneous mutations were found to partially reconstitute the helical regions. In addition, mutations at both sides of the stems, maintaining the structure but changing the nucleotide sequence, replicated efficiently. These observations, together with the sequence conservation, and the co-variations observed in different positions within S1 and S2, indicated a requirement of the bottom part of the SLA for viral replication. Deletion or substitution of the conserved U_62_U_63_ bulge between the helical regions S1 and S2, indicated that this element is essential for replication, and that at least one U residue in the bulge is required. Substitution of the UU bulge for an AA bulge gave rise to revertant viruses with a UA bulge, confirming the requirement of at least one U in that position. Deletion of the side stem loop abolished viral replication, however, changing the sequence of the stem, the sequence of the loop, or modifying the length of the stem resulted in RNAs that were able to replicate [14]. Regarding the helical region S3, substitutions disrupting three base pairings in the middle of S3 yielded viral RNAs that replicated as efficiently as the parental RNA. However, disruption of the closing base pairing at the top of S3 was lethal, suggesting that although S3 tolerates large variations, structural features of this region are important for viral replication. Substitution of nucleotides present at the top loop of SLA resulted in spontaneous mutations that rescued viral replication, suggesting an essential role of this element.

Footprinting studies carried out to investigate the interaction of NS5 with the viral 5′UTR RNA were recently reported [64]. Both NS5 and the RdRp domain showed protection of different regions of the SLA, including nucleotides at the top loop and the side stem loop. *In vitro* RNA binding assays using mutated RNAs with deletions of the top loop or the side stem loop showed a requirement of these two elements of the SLA for efficient ribonucleoprotein complex formation and polymerase activity *in vitro* [64]. Mobility shift assays were also used to investigate the elements of SLA involved in stable RNA-NS5 complex formation. The U bulge between S1 and S2 was found to be dispensable for NS5 binding or *in vitro* polymerase activity. The essential role of this U bulge in viral replication suggested an additional function of this element in infected cells. Substitutions of nucleotides present at the top loop of SLA inactivated the promoter for *in vitro* polymerase activity, which correlated with the lack of viral replication of RNAs carrying these mutations in transfected cells. Moreover, when the spontaneous changes obtained in the revertant viruses in cell culture were introduced in the SLA used for the *in vitro* assay, promoter activity was restored. Interestingly, RNA-protein binding studies with the top loop SLA mutants indicated that NS5 was able to form high affinity complexes that were inactive for RNA synthesis. This allowed dissociation of polymerase binding from polymerase activity, providing evidence for a post-binding activation of the polymerase [64].

A mutagenesis analysis of DENV NS5 to identify specific requirements for promoter-dependent polymerase activity was recently reported. In this study, a battery of 19 amino acid substitutions of basic residues present on the surface of the protein were designed in a recombinant protein and in the context of a DENV infectious clone [65]. Evaluation of polymerase activity using a non-specific poly(C) template or the viral 5′UTR revealed that mutation of amino acids present at the F1 region in the fingers domain of NS5 impaired SLA dependent initiation of RNA synthesis, without affecting elongation activity of the protein [65]. It has been proposed that interaction of specific nucleotides of the SLA promoter with NS5 induces a conformational change in the protein, which presumably involves the F1 motif, yielding a catalytically active enzyme. To understand in molecular detail the mechanism by which the SLA promotes RNA synthesis and to define the role of possible conformational changes in NS5 and/or the viral RNA during this process, three-dimensional structural studies of the complex formed between the SLA promoter and the viral RdRp will be necessary.

## 5. RNA Synthesis Silencing by the 3′SL Structure

The NS5 polymerase binds the SLA promoter and initiates *de novo* RNA synthesis copying from the 3′ end of the genome. Although 3′ nucleotides of the 3′SL must be recognized by the polymerase during the initiation process, the 3′UTR by itself does not function as template for *in vitro* RNA synthesis [15,66]. In addition, recent studies have shown that RNA molecules carrying an intact SLA promoter fused to the 3′SL structure are also inactive as templates for RNA synthesis [64]. Mutagenesis at the 3′ end of these molecules, together with *in vitro* polymerase assays, revealed that the large and stable stem of the 3′SL, represses RNA synthesis.

In the context of the viral genome, hybridization of the complementary sequences 5′-3′UAR induces conformational changes within the SLB at the 5′UTR, and the bottom half of the 3′SL at the 3′UTR (Figure 2). In this regard, it was proposed that hybridization of complementary sequences could play a dual role: (i) to bring the polymerase-SLA promoter near the 3′ end of the genome and (ii) to open the large stem of the 3′SL structure by 5′-3′UAR hybridization [64].

**Figure 3.**
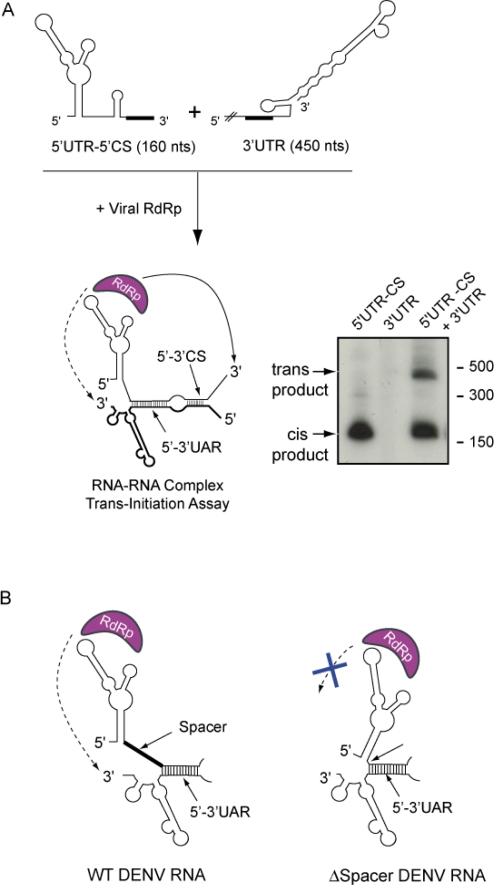
Representation of the *in vitro trans* initiation assay for RNA synthesis. (**a**) Formation of an RNA-RNA complex between the first 160 nucleotides of the viral genome (5′UTR-5′CS) and the viral 3′UTR allows the RNA-dependent RNA polymerase (RdRp) to initiate RNA synthesis at the 3′ end of both molecules, as indicated schematically in the figure. On the right, a representative native polyacrylamide gel shows the radiolabeled RNA products obtained after incubation of the viral RdRp with the templates described at the top of the figure. (**b**) Schematic representation of the role of a spacer sequence for RNA synthesis. The oligo(U) track spacer located between the SLA and the hybridized 5′-3′ UAR sequences allows accommodation of the RdRp to initiate RNA synthesis at the 3′UTR. In contrast, an RNA molecule carrying an intact SLA but a deletion of the oligo(U) spacer is unable to promote RNA synthesis in *trans*.

It has been reported that the DENV RdRp has a narrow template channel, which would only accommodate a 3′ end of an RNA in a single stranded form [60]. Thus, this structural constraint in the viral polymerase could explain the inhibitory effect of the 3′SL structure. The requirement of a conformational change within the 3′SL for RNA synthesis was recently investigated using a previously reported *trans* initiation polymerase assay [14,15,55,66]. In this assay, two different RNA molecules are included as templates: one containing the SLA followed by the 5′ cyclization elements, and the other one corresponding to the viral 3′UTR. These two molecules interact in solution to form an RNA-RNA complex resembling the ends of the genome in the circular conformation (Figure 3A). In these conditions, the polymerase copies the 5′RNA molecule in *cis* and the 3′UTR molecule in *trans*. The *trans* initiation of RNA synthesis was demonstrated to be absolutely dependent on RNA-RNA hybridization [17,55,58]. Using this assay with mutated 3′UTR molecules, it was shown that opening the 3′SL structure by UAR hybridization or by designing RNA molecules with unstructured 3′ ends, the inhibitory effect of the 3′SL structure was released. In addition, molecules that were able to form RNA-RNA complexes by artificially designed complementary sequences outside the 3′SL were useful to dissociate the two different roles proposed for 5′-3′UAR hybridization. Using these molecules, the requirement of an open bottom half of the 3′SL for RNA synthesis was confirmed. Previous studies have demonstrated that the 3′SL, including the structure of the large stem, is essential for DENV replication. Mutations that impair formation of the bottom half of the stem resulted in spontaneous mutations that restore the structure [67]. Together, the available information indicates that the 3′SL exists as structure during viral replication; however, this RNA structure must change in order to adopt an open conformation during the initiation of minus strand RNA synthesis.

## 6. Elements Downstream of the SLA that Modulate RNA Synthesis

Between the SLA structure and the 5′UAR sequence at the viral 5′UTR there is an oligo(U) track conserved in DENV and other flavivirus genomes (Figure 1B). Different studies using infectious clones, replicon systems, and *in vitro* assays showed that the oligo(U) track functions as spacer that enhances viral RNA synthesis [14]. Deletion of six Us downstream of the SLA in a DENV infectious clone resulted in viral attenuation. Replication of this virus was greatly delayed, and sequencing analysis of viral stocks obtained after several passages indicated that the 6U deletion was maintained. Also, it was demonstrated that the nucleotide sequence per se was not an important determinant for replication because replacement of 6Us by 6As yielded viruses with phenotypes that were indistinguishable from that of the parental virus. In addition, incorporation of the 6U deletion into a DENV replicon system indicated that translation of the RNA was unaffected while RNA synthesis was reduced about 40-fold [14].

The mechanism by which the oligo(U) track enhanced DENV RNA synthesis was investigated by testing the ability of the 5′UTR with or without the U track to interact with the 3′ end of the genome, and to promote polymerase activity using the *trans* initiation assay described above (Figure 3). These studies indicated that the long-range RNA-RNA interaction and the SLA promoter activity in *cis* were unaffected by the deletion. However, the *trans* initiation activity was seriously compromised when the oligo(U) spacer was shortened (Figure 3B). A direct correlation of RNA synthesis with the length of the U track was observed.

Recent footprinting studies indicated that binding of the DENV RdRp to the 5′UTR induces large conformational changes downstream of the SLA promoter, within the oligo(U) track spacer [64]. Nucleotides located at position 70 to 80 of the 5′UTR became highly susceptible to RNase A cleavage when bound to the viral polymerase [64]. These results together with the requirement of the oligo(U) spacer for RNA synthesis, provide evidence that polymerase binding to the viral 5′ end structure, in the context of the 5′-3′ RNA-RNA complex, leads to RNA conformational changes outside the SLA and the 3′SL.

## 7. Dynamic Conformations of the DENV Genome Are Necessary for RNA Synthesis

A sequence at the 5′ end of the 3′SL, which includes the 3′UAR cyclization element, forms a small hairpin (sHP) (Figure 2). Thus, nucleotides of the sHP fold into two alternative structures: they form an extended duplex in the circular conformation of the genome or fold locally into the sHP. Mutations in a DENV full-length RNA indicated that alteration of any of the two structures, the duplex or the sHP, impairs viral RNA replication [59]. The overlapping nature of these structures and the essential requirement of both for viral RNA synthesis indicated that the viral genome must exist in at least two different conformations during infection. Unlike well studied riboswitches in cellular RNAs, the importance of conformational changes in viral RNAs during infection is a new area of investigation [68,69]. How and why viral RNAs change their structure is still unclear. It is likely that host or viral proteins that interact with the RNA could participate in modulating this process. For instance, the RNA helicase activity of the viral protein NS3 is essential for genome replication, however, the mechanism by which this protein regulates the viral RNA structure is poorly understood. In addition, a number of host proteins have been reported to bind the viral RNA. For example, *in vitro* studies have demonstrated the binding of cellular proteins to different regions of flavivirus 3′UTRs. Binding of EF-1α, TIA, the related protein TIAR, PTB, YB-1, Mov34, human La autoantigen, and NF90 to the viral RNA has been demonstrated [70–79]. It is important to further investigate how binding of these cellular proteins to the viral RNA participates in viral replication.

**Figure 4.**
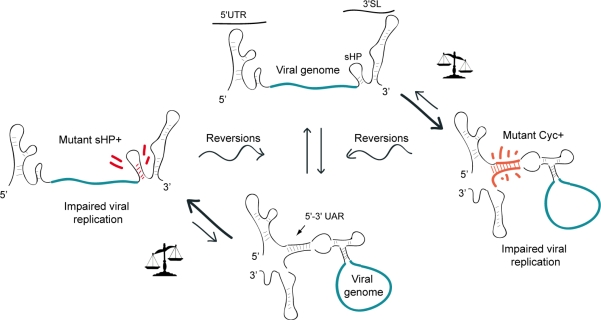
Representation of a model showing the requirement of a balance between different conformations of the DENV genome. Viruses carrying mutations that increase the stability of the circular or the linear form of the RNA, by increasing 5′-3′UAR complementarity (Mut Cyc+) or by stabilizing the sHP (Mut sHP+) respectively, evolve in culture incorporating spontaneous mutations that restore the relative stability of the two competing structures.

It is possible that the presence of competing structures in the viral genome could provide a way to modulate different RNA conformations. To test this idea in the DENV genome, the effect of mutations displacing the equilibrium towards the circular or the linear form of the RNA on viral replication was evaluated [59]. Mutations that increased the stability of the circular or the linear conformation of the genome impaired viral replication in cell culture. However, a wide variety of spontaneous mutations rescued viral replication. To obtain information of nucleotide changes at the 5′ and 3′ ends of the genome in the revertant viruses, the ends of the isolated genomes were ligated and sequenced simultaneously. In all the replicating viruses, the nucleotide changes tended to restore the wild type equilibrium between the competing structures. In this study, two types of spontaneous reversions were reported. In the first case, the stability of the structure altered by the mutation was restored. In the second type, spontaneous mutations stabilizing the competing structure were rescued. For instance, transfection of a mutant RNA with stabilized sHP structure resulted in revertant viruses that increased the 5′-3′UAR complementarity. This observation highlighted the importance of the relative stability between the two competing conformations rather than the absolute stability of each structure. Based on these studies it was proposed that a balance between at least two conformations of the DENV genome would be necessary for RNA replication and viral infectivity (Figure 4).

## 8. Future Prospects

A great deal has been learned in the last years about the role of RNA signals present in the DENV genome. However, mechanistic aspects that explain how these structures participate in the viral processes are still lacking. How does the SLA interact with the polymerase to promote RNA synthesis on the authentic 3′ end of the genome? How does the 3′SL change its conformation during the initiation process in the infected cell? What is the role of helicases and RNA chaperones in modulating the architecture of viral genomes? It is of great interest to define the interplay between the promoter SLA, the 3′SL, the viral NS5 polymerase, and RNA structures that work as enhancers of viral RNA synthesis. It is likely that these RNA signals interact with other RNA molecules and/or proteins. Therefore, effort towards identifying functionally relevant binders of these RNA structures will be necessary to fully understand their mechanism of action. Another important question is whether the functional structures identified as regulators of RNA synthesis are necessary for both minus and plus strand RNA amplification.

It has been widely accepted that flavivirus genome cyclization is essential for viral replication, however, recent observations support the idea that linear and circular conformations of the viral RNA co-exist and that dynamic RNAs are required for viral replication. This new view of the viral genome, as a flexible molecule, will help to understand the function of alternative RNA structures formed in different stages of the viral life cycle. Finally, because the DENV RNA acquires complex secondary and tertiary structures, the challenge is to study these high order structures in the context of the whole viral genome.
